# Machine learning methods evaluation for identification of cognitive phenotypes in multiple sclerosis and their MRI correlates

**DOI:** 10.3389/fnins.2026.1878685

**Published:** 2026-07-24

**Authors:** Patrycja Romaniszyn-Kania, Weronika Galus, Julia Wyszomirska, Katarzyna Zawiślak-Fornagiel, Oskar Bożek, Daniel Ledwoń, Damian Kania, Aleksandra Tuszy, Joanna Siuda, Andrzej W. Mitas

**Affiliations:** 1 Faculty of Biomedical Engineering, Silesian University of Technology, Zabrze, Poland; 2 Department of Neurology, Faculty of Medical Sciences in Katowice, Medical University of Silesia, Katowice, Poland; 3 Department of Neurology with the Stroke Subunit Prof. K. Gibinski University Clinical Center of Medical University of Silesia in Katowice, Katowice, Poland; 4 Department of Psychology, Faculty of Health Sciences in Katowice, Medical University of Silesia, Katowice, Poland; 5 Department of Radiology and Nuclear Medicine, Faculty of Medical Sciences in Katowice, Medical University of Silesia, Katowice, Poland; 6 Institute of Physiotherapy and Health Sciences, The Jerzy Kukuczka Academy of Physical Education in Katowice, Katowice, Poland

**Keywords:** cognitive impairment, cognitive profile modeling, machine learning, multiple sclerosis, neurodegenerative diseases

## Abstract

**Background:**

Cognitive impairment (CI) is common in multiple sclerosis (MS) yet poorly captured by conventional disability scales. Although neuropsychological assessment and magnetic resonance imaging (MRI) are routinely used separately, there is no simple clinically applicable framework integrating cognitive performance with structural brain changes to identify patients at increased risk of cognitive decline. Integrating neuropsychological testing with MRI-based atrophy metrics may yield clinically useful cognitive phenotypes with differential patterns of brain atrophy measures.

**Methods:**

Data were collected from 79 patients with multiple sclerosis (PwMS) who underwent comprehensive neuropsychological assessment and brain MRI. Neuropsychological variables were subjected to a feature selection procedure based on variance and quartile coefficient of dispersion filtering, followed by Pearson correlation and mutual information (MI) analyses to generate reduced feature sets. These feature sets were used as input for unsupervised clustering with the Partitioning Around Medoids (PAM) algorithm to identify cognitive phenotypes. Differences between the resulting groups in the degree of brain atrophy measures were subsequently evaluated using appropriate statistical tests—one-way ANOVA or the Kruskal–Wallis test. *Post hoc* analysis was performed using a pairwise *t*-test, Welch's *t*-test, or Wilcoxon test with the Holm-Bonferroni correction, depending on the data distribution and variance.

**Results:**

The feature selection procedure based on variance and mutual information identified neuropsychological features that were subsequently used for clustering. Based on these features, the PAM algorithm identified three distinct groups of PwMS that differed in their clinical characteristics, degree of brain atrophy measures, and cognitive phenotype, ranging from preserved cognition to global cognitive impairment.

**Conclusion:**

Three cognitive phenotypes with differential patterns of brain atrophy measures integrate neuropsychological testing with MRI measures into a clinically applicable framework that may help bridge the gap between structural imaging findings and everyday cognitive assessment in PwMS. This approach may improve screening, enable earlier detection of CI, improve monitoring, and provide valuable information for rehabilitation planning.

## 1 Introduction

Multiple sclerosis (MS) is a chronic immune-mediated central nervous system disorder characterized by co-occurring inflammatory, demyelinating, and neurodegenerative processes leading to progressive disability (Filippi et al., 2018). Although MS presents heterogeneously, cognitive impairment (CI) is a major clinical issue, often appearing early and affecting quality of life, work functioning, and rehabilitation outcomes. These deficits are not well-captured by traditional physical disability measures, such as the Expanded Disability Status Scale (EDSS), highlighting the need for systematic and sensitive cognitive assessments (Kurtzke, 1983).

Magnetic resonance imaging (MRI) is the gold standard for diagnosing and monitoring MS (Montalban et al., 2025), enabling assessment of lesion dissemination in space and time and measurement of brain atrophy (Thompson et al., 2018). Metrics such as lesion load, cortical and subcortical volumes, and ventricular enlargement provide insight into disease mechanisms (Cagol et al., 2022). However, standard MRI measures do not fully account for cognitive variability among patients, underscoring the growing importance of multimodal approaches (Zawiślak-Fornagiel et al., 2026).

Integrating neuropsychological data with morphometric MRI measures may provide a more accurate reflection of a patient's condition than single-modality analyses. Combining multimodal assessment with exploratory analytic methods also supports the identification of new MS-related biomarkers. Increasingly, MS progression and patient status are modeled using approaches from classical regression to deep learning (Yousef et al., 2024).

The approach presented by Liang et al. (2024) aims to define the cognitive profiles of people with MS and to determine disability progression based on MRI data and the k-means algorithm. Pourzinal et al. (2020) used neuropsychological data and machine learning methods to define cognitive profiles of patients with Parkinson's disease. De Meo et al. (2021) defined clinical and MRI features for each phenotype in patients with MS, based on a battery of neuropsychological tests, MRI data, regression algorithms, and mixed models. In a study by Rimkus et al. (2024) based on cognitive assessment and other psychological data, the k-means method was used to group MS patients. Plow and Gunzler (2022) used exploratory structural equation modeling to examine relationships among fatigue, depression, and cognitive impairment in MS. Alirezaei et al. (2021) applied simple and multivariate regression to identify predictors of cognitive performance. Mancini et al. (2016) combined graph theory and factor analysis to define MRI-based factors and their structural–functional correlates. Exploratory factor analysis was used to develop a model of gait disorders in patients with MS (Angelini et al., 2021).

Therefore, the aim of this study was to address the clinical challenge of linking cognitive dysfunction to structural brain changes in MS using a clinically feasible and interpretable framework. By combining feature reduction methods with unsupervised machine learning, we aimed to identify key neuropsychological measures and define cognitive phenotypes with differential patterns of brain atrophy measures associated with different degrees of neurodegeneration and cognitive impairment.

## 2 Materials and methods

### 2.1 Participants

A total of 79 patients receiving care at the Department of Neurology and the Neurological Outpatient Clinic of the University Clinical Center, Prof. Gibiński, Medical University of Silesia in Katowice, were recruited for this study. Detailed characteristics of SM patients were shown in Table 1.

**Table 1:** Characteristics of the MS patient group (mean ± standard deviation, for EDSS median ± quartile deviation.

Feature	RRMS (*n* = 64)	PPMS (*n* = 10)	RRMS RES (*n* = 5)
Age, years	42.61 (11.06)	55.30 (7.30)	36.20 (13.01)
Female, %	73.44	80.00	80.00
Disease duration, year	11.91 (7.97)	10.90 (3.93)	11.80 (8.44)
Patients with more than 12 years of education, %	55	6	5
EDSS	2.00 (1.05)	5.25 (0.25)	1.50 (1.25)
MoCA	26.96 (3.37)	27.00 (2.14)	27.20 (0.84)
BDI	11.47 (10.08)	8.75 (5.76)	5.60 (4.16)

RRMS, relapsing-remitting multiple sclerosis; PPMS, primary progressive multiple sclerosis; RRMS RES, relapsing-remitting rapidly evolving severe multiple sclerosis; EDSS, expanded disability status scale; MoCA, Montreal cognitive assessment; BDI, Beck's depression inventor.

All participants provided informed consent. Inclusion and exclusion criteria were presented in Table 2.

**Table 2:** Inclusion and exclusion criteria for patients recruited for the study.

Inclusion criteria	Exclusion criteria
Age ≥ 18 years	MS relapse within the preceding 6 weeks
A diagnosis of relapsing–remitting multiple sclerosis (RRMS), secondary progressive multiple sclerosis (SPMS) and primary progressive multiple sclerosis (PPMS)	Coexisting neurological or psychiatric disorders, including depression with clinical worsening in the prior 4 weeks, cognitive impairment at the level of severe dementia
EDSS ≤ 6.5	–
Ongoing disease-modifying therapy (DMT)	Treatment outside standard DMT (e.g., mitoxantrone, cyclophosphamide)

MS, multiple sclerosis; RRMS, relapsing-remitting multiple sclerosis; PPMS, primary progressive multiple sclerosis; SPMS, secondary progressive multiple sclerosis; EDSS, expanded disability status scale; DMT, disease modifying therapy.

At baseline, a detailed medical history was obtained, and a neurological examination, including EDSS scoring, was performed. All participants subsequently underwent a comprehensive neuropsychological assessment administered by an experienced clinical neuropsychologist. A contrast-enhanced brain MRI was acquired within a maximum of 6 months of the baseline evaluation, as detailed below.

The Research Ethics Committee of the Medical University of Silesia approved the study protocol, which was conducted in accordance with the principles outlined in the Declaration of Helsinki (BNW/NWN/BNW/NWN/0052/KB1 /45/24).

### 2.2 MRI examination

All participants underwent brain MRI in accordance with the Recommendations of the Polish Medical Society of Radiology and the Polish Society of Neurology (Sąsiadek et al., 2020) using a GE SIGNA Artist 1.5T scanner. The volumetric datasets included a pre-contrast T1-weighted MP-RAGE and a T2-weighted FLAIR FS CUBE. DICOM images were processed with SCENIC filtering. Brain segmentation and atrophy assessment were performed with FreeSurfer v7.4.1 (Fischl, 2012), with volumes normalized to the estimated total intracranial volume (eTIV). The analysis encompassed gray and white matter, cerebrospinal fluid, and subcortical structures (ventricles, thalamus, hippocampus, cerebellum). The segmentation masks were initially reviewed by an experienced radiologist. To ensure the reproducibility of the measurements and to avoid operator-dependent variability, the segmentation results were not manually adjusted. White-matter lesion burden was quantified using the Lesion Segmentation Toolbox (Wiltgen et al., 2024).

### 2.3 Cognitive assessment

We selected neuropsychological assessment tools that cover a broad spectrum of cognitive domains, encompassing both auditory and visual modalities, as well as graphomotor tasks. Preference was given to instruments commonly used in previous research and recommended for evaluating cognitive functions in patients with multiple sclerosis. We used the following neuropsychological tests: The Montreal Cognitive Assessment (MoCA) for general cognitive screening (Gierus et al., 2015), following by Symbol Digit Modalities Test (SDMT) (Iverson et al., 2020), Color Trails Test (CTT) (Łojek and Stańczak, 2012), California Verbal Learning Test (CVLT) (Łojek and Stańczak, 2010), Benton Visual Retention Test (BVRT) (Jaworowska et al., 2019), Verbal Fluency Test (VFT) (Gawda and Szepietowska, 2021) and Victoria Stroop Test (VST) (Troyer et al., 2006). Raw test scores were converted into standardized clinical scores to facilitate the identification of individuals with deficits in specific cognitive domains. All information regarding the assessment tools, scales, and cut-off for impairment is included in the Table 3.

**Table 3:** Neuropsychological tests and subscales used to assess cognitive state with the cut-off for impairment in the given scale.

Neuropsychological tools	Scales and indicators	Cut off scores for impairment
Symbol Digit Modalities Test (SDMT) assesses processing speed, executive function, working memory, sustained attention, and visuomotor coordination, sensitive to cognitive slowing in neurological conditions		Compared with means and standard deviation, < 1.5 SD
Color Trails Test (CTT) assessing efficiency of visual attention, processing speed, and executive functions	CTT 1	< 16th percentile
	CTT 2	
	Interference Index (CTT.II)	
California Verbal Learning Test (CVLT) assessing verbal learning and recall strategies, short- and long-term memory, and executive functions	Total Recall Trails (TRT)	< 4th sten scores
	Short-Delay Free Recall (SDFR)	
	Long-Delay Free Recall (LDFR)	
	Intrusions Errors (IE)	
	Discrimination Index (DI)	
	Total Errors (TE)	
Benton Visual Retention Test (BVRT) assessing short-term visual memory, perception, and visual-constructive abilities	Number of Correct Reproductions (NCR)	Category: low score
	Number of Errors (NE)	< 4th sten scores
Verbal Fluency Tests (VFT) assess many cognitive functions simultaneously, including processing speed, executive functions, and semantic and lexical memory	Phonemic Fluency (PF)	< 4th sten scores
	Semantic Fluency (SF)	
	Verb Fluency (VF)	
	Low-difficulty Tasks (LT)	
	High-difficulty Tasks (HT)	
	Emotional Fluency (EF)	
	Total Score (TS)	
Victoria Stroop Test (VST) assessment of processing speed, attention, executive function, cognitive, and resistance to interference	Dot condition (VST I)	< 7 Scaled Scores
	Word condition (VST I)	
	Interference Index (VST.III)	

SDMT, Symbol Digit Modalities Test; CTT, Color Trails Test; CTT1, first part of CTT; CTT2, second part of CTT; CTT.II - CTT Interference Index; CVLT, California Verbal Learning Test; CVLT.TRT, Total Recall Trials; CVLT.SDFR, Short-Delay Free Recall; CVLT.LDFR, Long-Delay Free Recall; CVLT.IE, Intrusions Errors; CVLT.TE, Total Errors; CVLT.DI, Discrimination Index; BVRT, Benton Visual Retention Test; NCR, Number of Correct Reproductions; NE, Number of Errors; VFT, Verbal Fluency Test; VFT.PF, Phonemic Fluency; VFT.SF, Semantic Fluency; VFT.VF, Verb Fluency; VFT.LT, Low-difficulty Tasks; VFT.HT, High-difficulty Tasks; VFT.EF, Emotional Fluency; VFT.TS, Total Score; VST, Victoria Stroop Test; VST.I, Dot condition; VST.II, Word condition; VST.III, Interference Index.

A comprehensive neuropsychological assessment was administered and scored by a clinical neuropsychologist. The testing was conducted under standardized conditions: individually, during a single session, with the identical order of test administration maintained for all participants.

Records from 57 patients with complete neuropsychological and imaging data was used for detailed analysis. The final number of cases is due to the exclusion of ten patients with PPMS and a further 12 patients due to incomplete psychological data or lack of MRI examinations (11 patients with RRMS, and one patient with RRMS-RES). Patients with PPMS were excluded from the main analysis because PPMS represents a clinically and radiologically distinct MS phenotype, often associated with older age, a different MRI lesion distribution with relatively greater spinal cord involvement, and a higher risk of age- or comorbidity-related cognitive impairment, which could increase sample heterogeneity and confound neuropsychological-imaging associations.

### 2.4 Feature selection

Various feature selection methods were used to identify consistent cognitive profiles in patients with MS. According to Table 3, 22 neuropsychological variables derived from SDMT, CTT, CVLT, BVRT, VFT and VST were initially considered as candidate features for clustering. Because the sample size was relatively small compared with the number of neuropsychological variables, an initial feature reduction step was performed to eliminate variables with low information content and to reduce redundancy before clustering. In the first step of the feature reduction analysis, it was decided to determine the variance for each psychological variable, which allows for the removal of variables with minimal variability. Based on the obtained values, a threshold was established to eliminate features from further analysis that do not differentiate observations and may introduce noise (i.e., the smaller the variance, the less information the feature contributes to the potential model) (Han and Yu, 2012). The quartile coefficient of dispersion (QCD) was used as an alternative method of initial elimination, as this algorithm is robust to outliers and non-standard distributions, detecting features with low relative variability (Bonett, 2006). The threshold was determined based on inspection of the distribution of QCD values across all cognitive variables before clustering.

**Figure 1:**
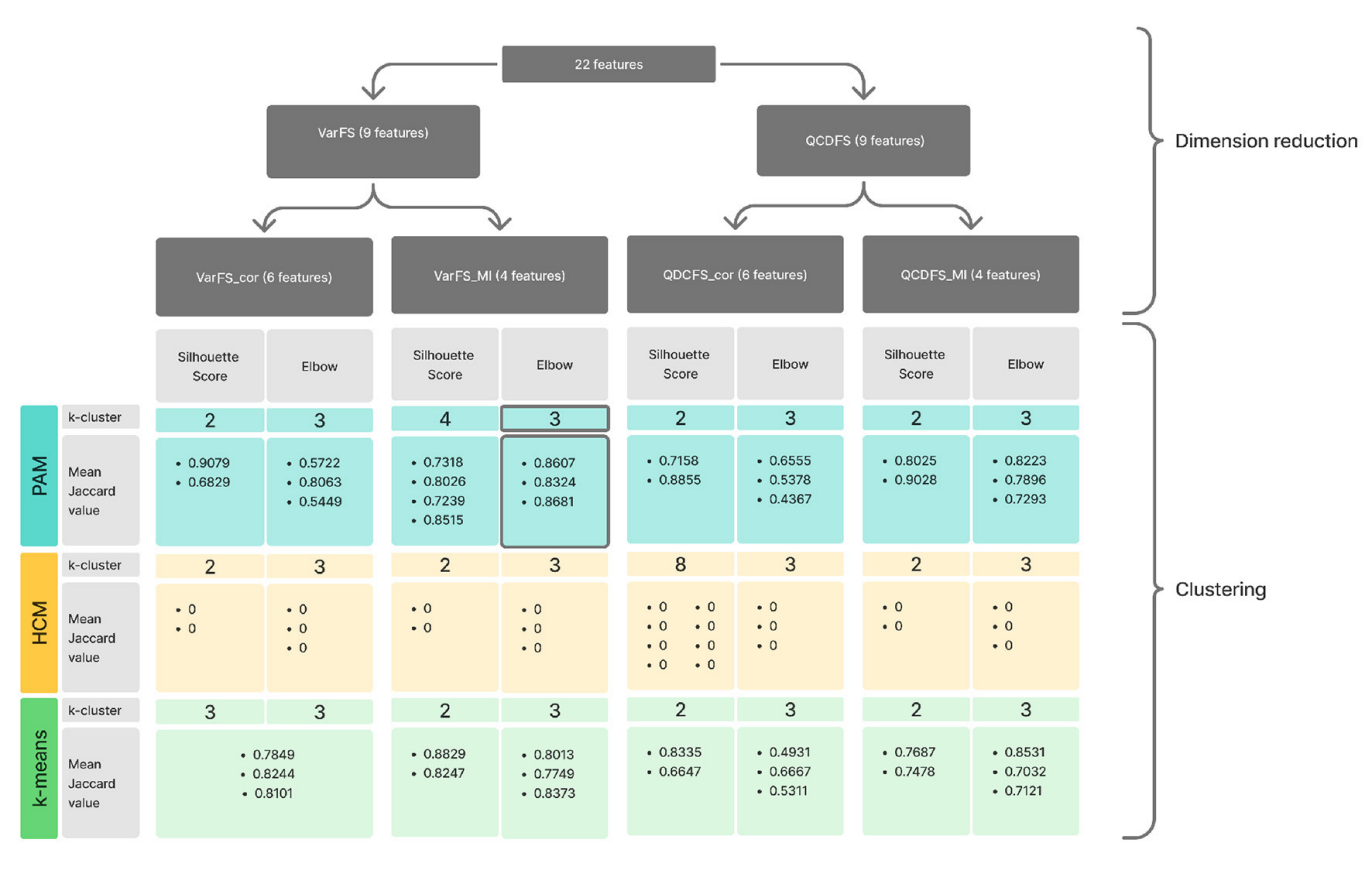
Flow of the presented feature reduction methods and clustering algorithms, including the number of features retained at each stage of the analytical workflow. The diagram summarizes the analytical pipeline from the initial neuropsychological variables through feature selection to the clustering procedures used to identify cognitive phenotypes.

Next, for two independent datasets (after reduction (1) by variance and (2) by the QCD algorithm), a Pearson correlation analysis of the features was performed (Benesty et al., 2009). For each correlated pair, one variable was removed according to predefined feature selection criteria—this elimination was performed until the correlation coefficients (*r*) between the variables reached *r* < 0.7. This allowed for a further reduction of the feature set. An alternative method to correlation analysis was the use of the Mutual Information (MI) algorithm, which detects any relationship between variables, not just linear ones, as in the case of correlations (Huang et al., 2024). Variables with MI coefficients less than 0.2 were removed from further analysis. The objective of the feature-selection procedure was to derive reduced feature sets that were subsequently used as input for the clustering algorithms – four independent feature sets (FS). The workflow of feature reduction methods was shown in Figure 1, marked as dimension reduction.

### 2.5 Cluster analysis

To determine the optimal number of clusters for the most differentiating features elbow methods, including the calculation of within-cluster sum of squares (WCSS) (Kuraria et al., 2018) and Silhouette Score (Shahapure and Nicholas, 2020), were applied for 20 consecutive iterations. The random number generator seed was set. The clustering was performed for a specified number of clusters using: (1) Partition Around Medoids (PAM or k-Medoids), which is not sensitive to outliers and reduces noise, as well as minimizes the sum of differences between data objects (Park and Jun, 2009), (2) Hierarchical Clustering Methods (HCM) based on the aggregation or division of multi-level clusters (Murtagh and Contreras, 2012), and (3) k-Means, which iteratively assigns each data point to the nearest centroid (Sinaga and Yang, 2020). The Bootstrapping method was used to verify the stability of the models (Tsamardinos et al., 2018). Cluster solutions with an appropriate number of clusters were evaluated. Stability was assessed using bootstrap resampling with 1,000 iterations and quantified using the Jaccard index between the original and resampled cluster assignments. To ensure reproducibility, a fixed random seed (123) was applied throughout the clustering procedure.

### 2.6 Statistical analysis

The class division obtained using unsupervised clustering allowed for statistical analysis of the entire dataset, excluding the variables used to build the model. For continuous variables, normality was assessed using the Shapiro–Wilk test. Depending on the distribution of the data and homogeneity of variance, differences between groups were evaluated using either one-way ANOVA or the Kruskal–Wallis test. *Post hoc* analysis was performed using a pairwise *t*-test, Welch's *t*-test, or Wilcoxon test with the Holm-Bonferroni correction, depending on the data distribution and variance. The significance level was set to α = 0.05. The analysis was performed using R 4.5.0 (R Core Team) and RStudio (Posit, PBC, Vienna, Austria) 2024.12+536.

## 3 Results

### 3.1 Feature selection

**Figure 2:**
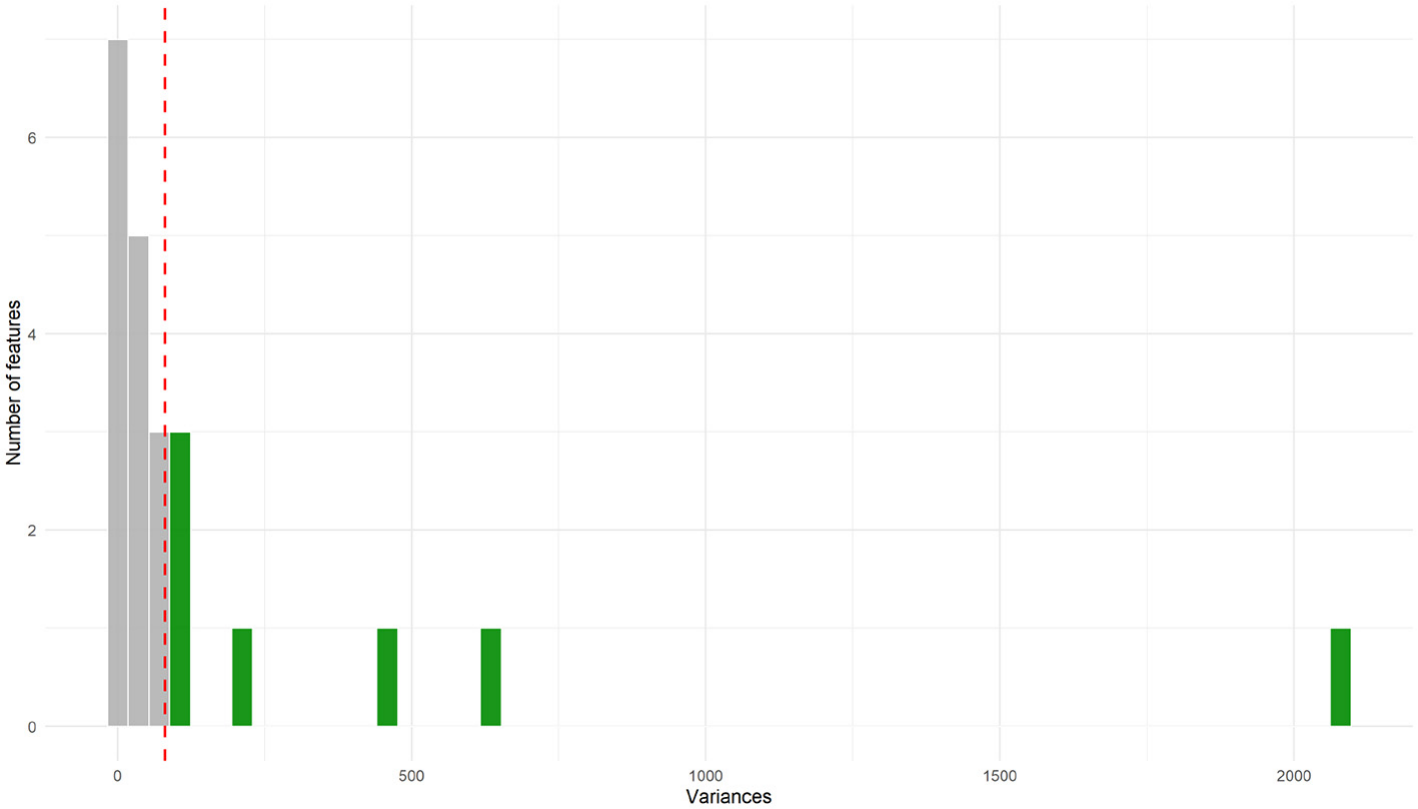
Variance distribution for the analyzed neuropsychological features. The dashed red line indicates the empirically determined variance threshold used for feature reduction. Features retained for subsequent analyses are shown in green, whereas excluded features are shown in gray.

The first step in psychological data selection involved feature reduction by determining the variance for each feature independently. The range of values was from 0.2240 for CTT Interference Index to 2,079.5457 for CTT 1. Based on the analysis of the variance distribution (Figure 2), a cut-off threshold of 80 was determined. Features above the threshold were included in further analysis—nine out of 22 initial psychological features (marked in green on the Figure 2)—as variables that contributed a lot of information to the model, indicating diversity of values.

**Figure 3:**
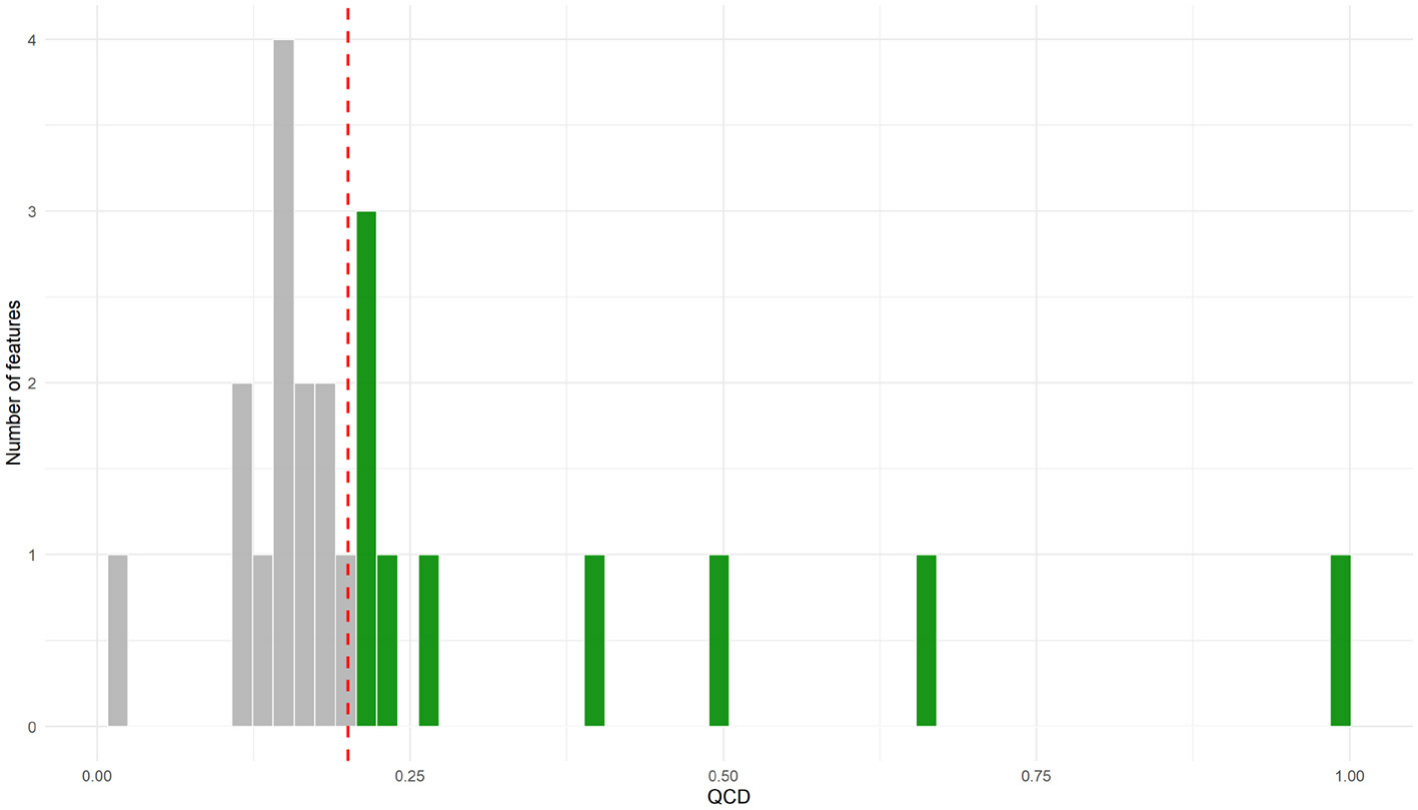
QCD distribution for the analyzed neuropsychological features. The dashed red line indicates the empirically determined QCD threshold used for feature reduction. Features retained for subsequent feature selection steps are shown in green, whereas excluded features are shown in gray.

The QCD values range from 0 to 1, with variables above the cut-off threshold of 0.2 included in further analysis—again nine out of 22 (Figure 3).

**Figure 4:**
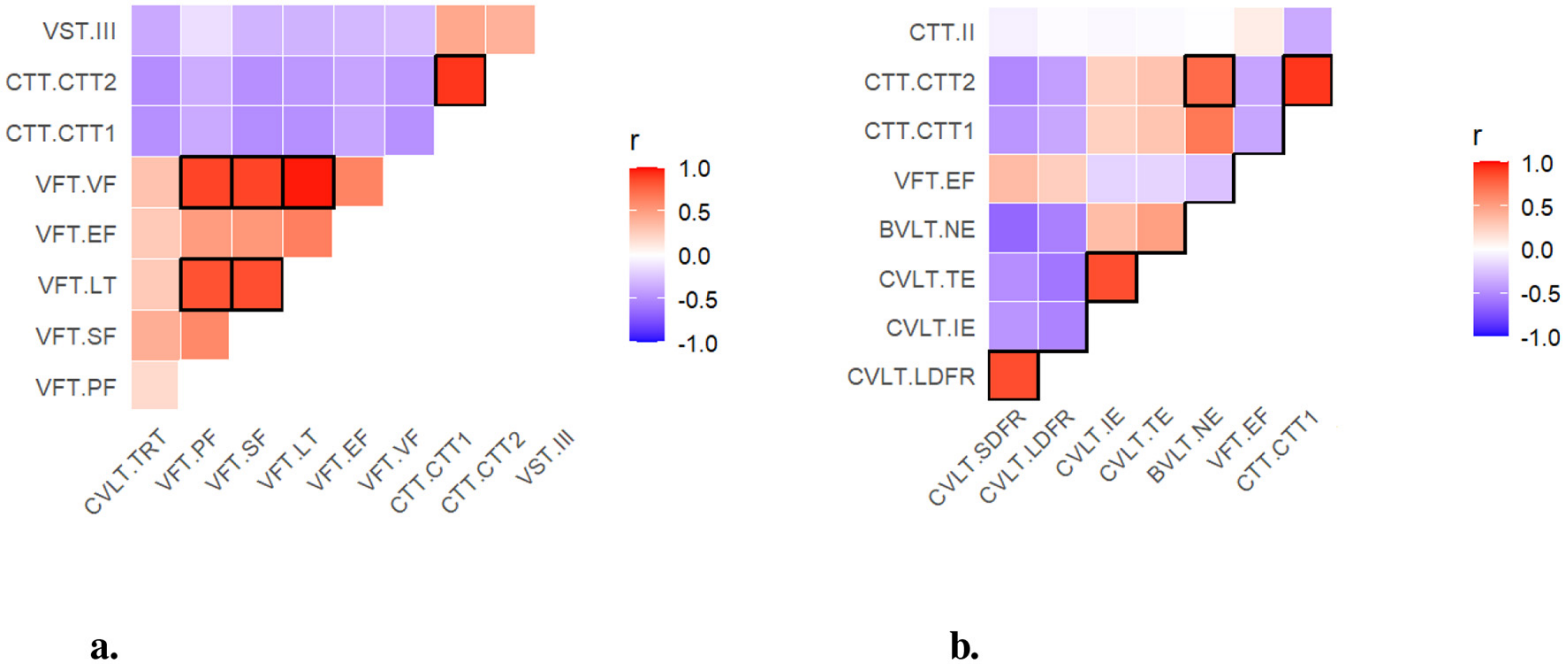
Correlation matrices for the two independent feature sets (a Var_cor and b QCD_cor). The heatmaps illustrate pairwise Pearson correlation coefficients between the retained neuropsychological features after feature reduction. Variables with absolute correlation coefficients exceeding the predefined threshold were considered for removal to reduce redundancy prior to clustering. **(a)** Correlation matrix for the Var_cor feature set. **(b)** Correlation matrix for the QCD_cor feature set. SDMT, Symbol Digit Modalities Test; CTT, Color Trails Test; CTT1, first part of CTT; CTT2, second part of CTT; CTT.II - CTT Interference Index; CVLT, California Verbal Learning Test; CVLT.TRT, Total Recall Trials; CVLT.SDFR, Short-Delay Free Recall; CVLT.LDFR, Long-Delay Free Recall; CVLT.IE, Intrusions Errors; CVLT.TE, Total Errors; CVLT.DI, Discrimination Index; BVRT, Benton Visual Retention Test; NCR, Number of Correct Reproductions; NE, Number of Errors; VFT, Verbal Fluency Test; VFT.PF, Phonemic Fluency; VFT.SF, Semantic Fluency; VFT.VF, Verb Fluency; VFT.LT, Low-difficulty Tasks; VFT.HT, High-difficulty Tasks; VFT.EF, Emotional Fluency; VFT.TS, Total Score; VST, Victoria Stroop Test; VST.I, Dot condition; VST.II, Word condition; VST.III, Interference Index.

Next, based on multiple Pearson correlation analyses, another three (different) variables were eliminated from both the VarFS and QCDFS sets. The result of the initial correlation of the set of features is shown below (Figure 4).

**Figure 5:**
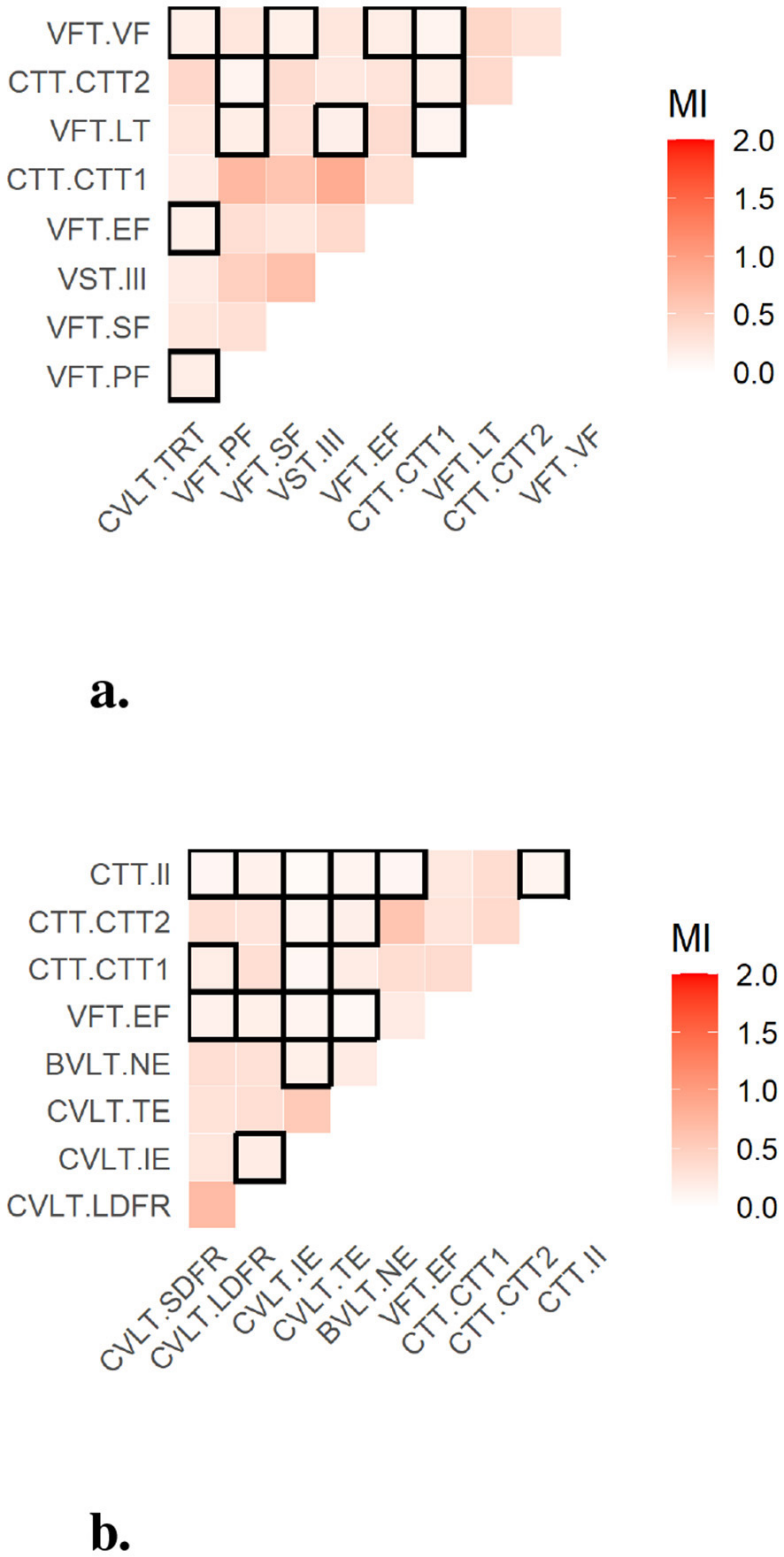
Mutual information matrices for the two independent feature sets (a Var_MI and b QCD_MI). The heatmaps show pairwise mutual information values between neuropsychological features after variance- or QCD-based filtering. Features with mutual information values below the predefined threshold were excluded from further analyses. **(a)** Mutual information matrix for the Var_MI feature set. **(b)** Mutual information matrix for the QCD_MI feature set. SDMT, Symbol Digit Modalities Test; CTT, Color Trails Test; CTT1, first part of CTT; CTT2, second part of CTT; CTT.II, CTT Interference Index; CVLT, California Verbal Learning Test; CVLT.TRT, Total Recall Trials; CVLT.SDFR, Short-Delay Free Recall; CVLT.LDFR, Long-Delay Free Recall; CVLT.IE, Intrusions Errors; CVLT.TE, Total Errors; CVLT.DI, Discrimination Index; BVRT, Benton Visual Retention Test; NCR, Number of Correct Reproductions; NE, Number of Errors; VFT, Verbal Fluency Test; VFT.PF, Phonemic Fluency; VFT.SF, Semantic Fluency; VFT.VF, Verb Fluency; VFT.LT, Low-difficulty Tasks; VFT.HT, High-difficulty Tasks; VFT.EF, Emotional Fluency; VFT.TS, Total Score; VST, Victoria Stroop Test; VST.I, Dot condition; VST.II, Word condition; VST.III, Interference Index.

Another algorithm used to analyse non-linear relationships between variables was to determine the Mutual Information value for each psychological feature combination in each dataset (VarFS, QCDFS) independently (Figure 5). Based on this, the number of features with MI values less than 0.2 was reduced again. For VarFS, five features were removed, while for QCDFS, five other features were removed.

### 3.2 Cluster analysis

The Elbow method and the Silhouette Score were used to determine the optimal number of *k* clusters in 20 consecutive iterations for each of the four psychological feature sets independently. Each time, successive unsupervised learning methods, such as PAM, HCM, and k-means, were used. Then, cluster stability was evaluated for all candidate solutions (2–4 clusters) using bootstrap resampling. Stability was quantified using the Jaccard index. Figure 1 presents the optimal number of clusters identified for each dataset and clustering method, together with the corresponding Jaccard index values. Based on the bootstrap stability analysis, the PAM algorithm was applied to the VarFS_MI dataset, and a three-cluster solution was selected as the final model (Jaccard index = 0.86).

**Figure 6:**
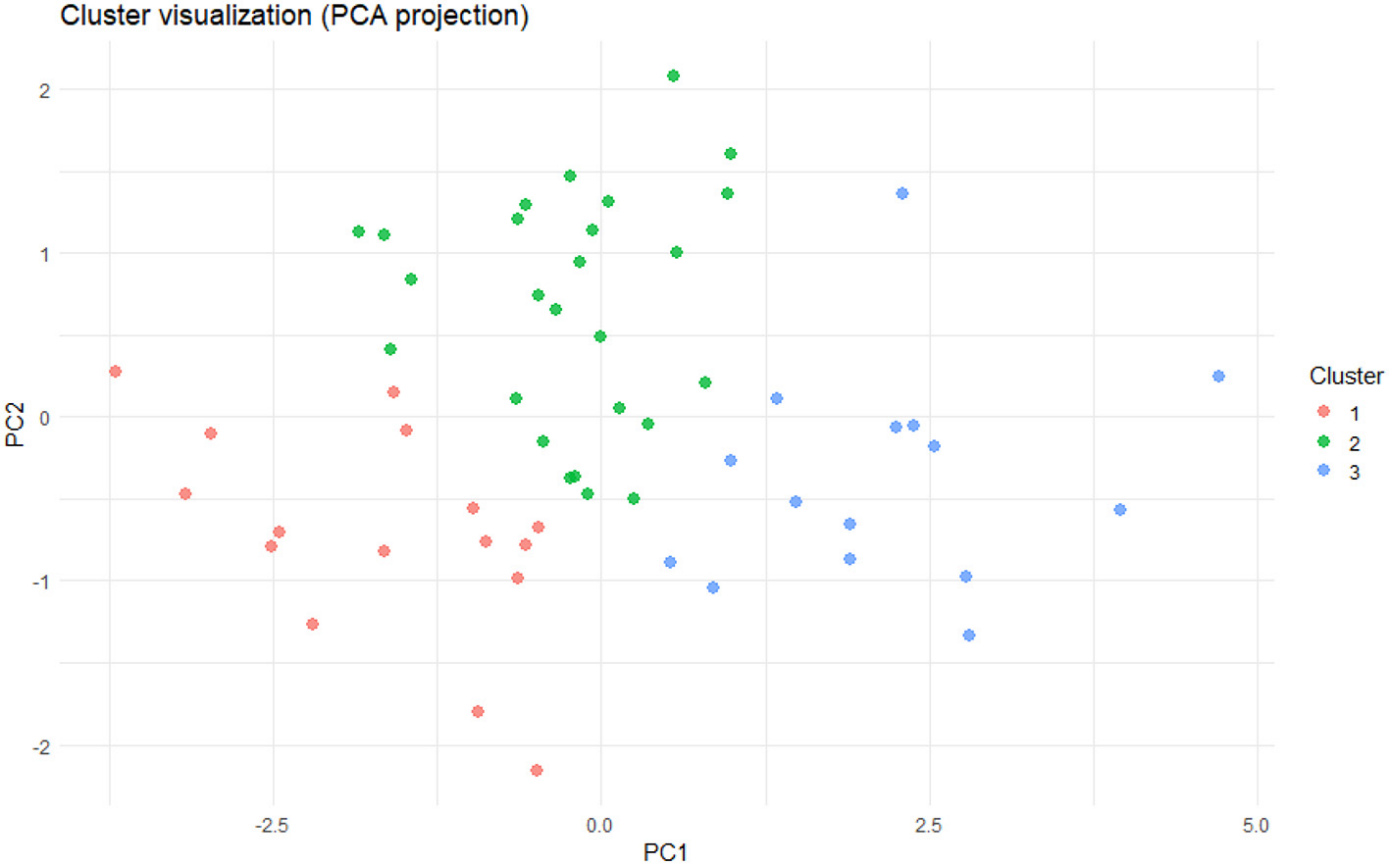
PCA projection of the clusters identified by the PAM algorithm. The figure illustrates the separation of the newly defined cognitive phenotypes in the reduced two-dimensional feature space based on the selected neuropsychological features.

Based on the bootstrapping results, the PAM algorithm was selected, with the set of features divided into three clusters after reduction using variance and MI (VarFS_MI) for clustering the subjects into newly defined groups. Patient groups were obtained in proportions of 16:26:15 (Figure 6).

**Figure 7:**
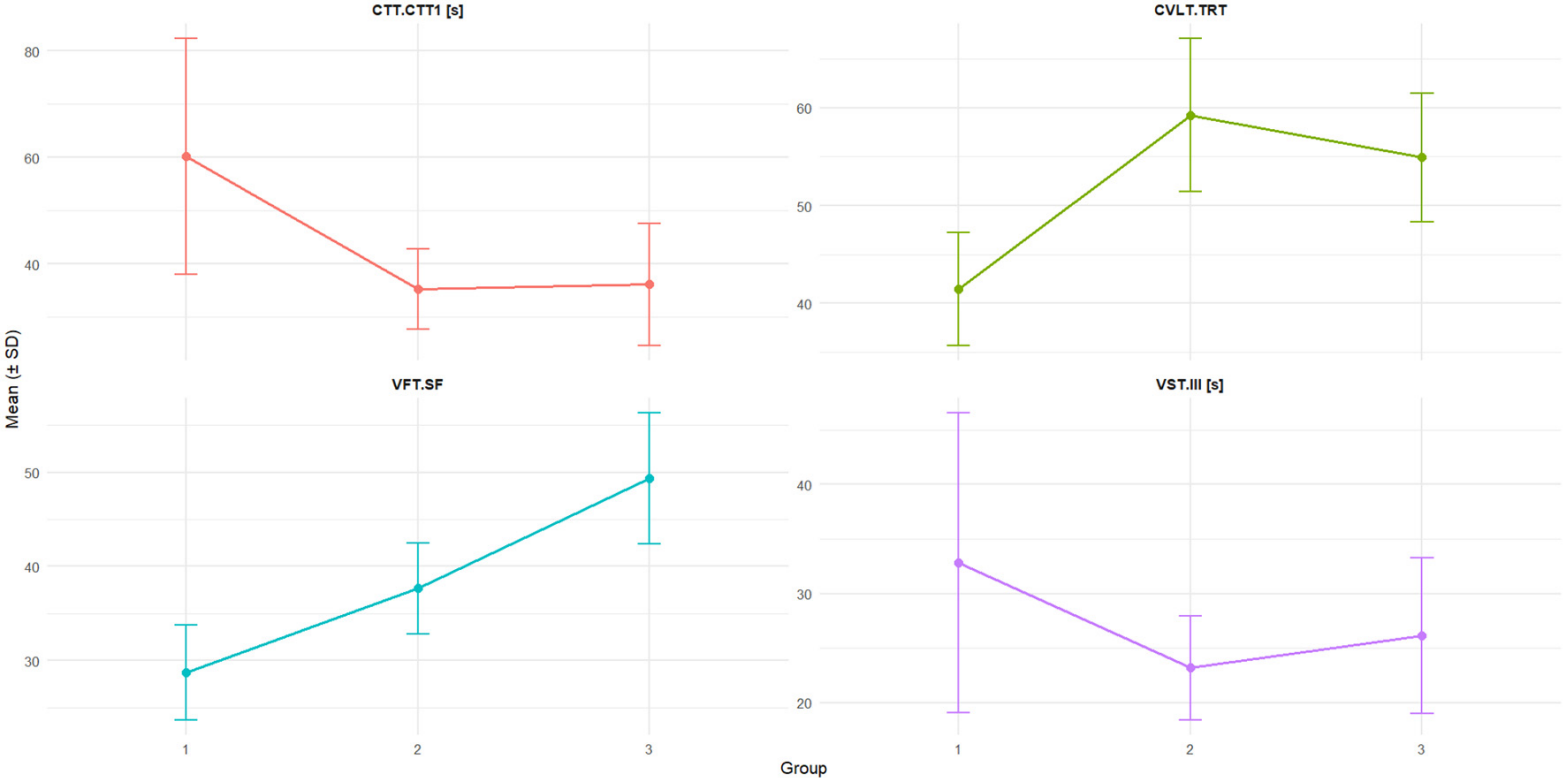
Mean values (± SD) of the neuropsychological features included in the VarFS_MI dataset for each newly identified patient group. The plot illustrates the differences in the retained features used for clustering and highlights the characteristic cognitive profiles of the identified groups.

Table 4 presents the clinical characteristics of the newly defined patient groups. Figure 7 illustrates the scores of neuropsychological variables that form the basis for group division.

**Table 4:** Clinical and demographic characteristics of the groups (mean ± standard deviation, for EDSS median ± quartile deviation).

Feature		Group 1 (*n* = 16)	Group 2 (*n* = 26)	Group 3 (*n* = 15)	*p*-value	Effect size
Age, years		47.44 (10.28)	39.65 (11.12)	46.20 (11.66)		
Female, %		68.75	73.08	86.67		
MS type	RRMS PPMS RRMS RES	16 0 0	24 0 2	13 0 2		
Disease duration, year		16.19 (7.88)	10.27 (8.11)	11.87 (6.93)		
Patients with more than 12 years of education, %		75.00	69.23	80.00		
EDSS		3.50 (0.50)	2.00 (0.25)	2.00 (0.87)	0.0017	η^2^ = 0.255
MoCA		23.69 (4.64)	27.85 (1.49)	28.75 (1.06)	0.0022	η^2^ = 0.381
BDI		13.87 (12.32)	10.35 (8.92)	5.93 (3.77)		

RRMS, relapsing-remitting multiple sclerosis; PPMS, primary progressive multiple sclerosis; RRMS RES, relapsing-remitting rapidly evolving severe multiple sclerosis; EDSS, expanded disability status scale; MoCA, Montreal cognitive assessment; BDI, Beck's depression inventor.

For the analyzed groups of patients and the new group division, a *post-hoc* analysis was performed to verify their clinical condition. Group 1 achieved a significantly higher (the highest one) mean EDSS score (3.00) compared to group 2 (the lowest score, 1.75), which indicates a clinically diverse distribution of patients. In addition, the number of impaired domains also differed significantly between the analyzed groups. Group 1 had the highest number of impaired domains (2.12), which was significantly higher than that of group 2 (0.42) and group 3 (0.53).

### 3.3 Neuropsychological profile of the identified groups

In the neuropsychological test results, analyzing *post-hoc* comparisons between groups, the most distinctive group was group 1 (Table 5). They obtained the lowest SMDT score, which was significantly different from that of group 2 (*p* = 0.004) and group 3 (*p* = 0.002). In the case of the CTT-2 result analysis, group 1 took the longest time to complete the test, significantly longer than the fastest group 3 (*p* < 0.001) and group 2 (*p* < 0.001).

**Table 5:** Mean results of individual tests and standard deviations for newly defined groups (mean ± standard deviation).

Neuropsychological tests	Feature	Group 1 (*n* = 16)	Group 2 (*n* = 26)	Group 3 (*n* = 15)	*p*-value	Effect size
Symbol Digit Modalities Test		30.00 (6.88)	49.54 (12.03)	44.83 (5.31)	< 0.001	ϵ^2^ = 0.45
Color Trails Test	CTT 2 [s]	109.57 (22.20)	70.30 (9.29)	70.00 (13.38)	< 0.001	η^2^ = 0.60
	II	0.91 (0.33)	1.05 (0.35)	1.06 (0.49)		
California Verbal Learning Test	SDFR	8.50 (2.50)	12.65 (2.28)	11.93 (2.71)	< 0.001	ϵ^2^ = 0.35
	LDFR	8.23 (1.69)	13.35 (2.02)	12.13 (2.77)	< 0.001	ϵ^2^ = 0.45
	IE	6.32 (6.80)	1.77 (2.81)	1.71 (2.33)	< 0.001	ϵ^2^ = 0.46
	DI	89.78 (4.84)	97.64 (2.50)	97.08 (2.42)	< 0.001	ϵ^2^ = 0.47
	TE	10.69 (6.95)	4.58 (3.88)	6.27 (4.62)	0.0122	η^2^ = 0.21
Benton Visual Retention Test	NCR	6.06 (1.69)	8.31 (1.16)	8.00 (1.51)	0.0003	ϵ^2^ = 0.29
	NE	5.87 (2.47)	2.08 (1.47)	3.13 (2.23)	< 0.001	ϵ^2^ = 0.37
Verbal Fluency Test	PF	23.69 (7.91)	24.85 (4.52)	39.20 (2.23)	< 0.001	η^2^ = 0.51
	VF	15.81 (4.56)	15.31 (4.02)	23.80 (5.88)	< 0.001	η^2^ = 0.39
	LT	45.45 (11.37)	51.40 (6.34)	75.13 (9.01)	< 0.001	η^2^ = 0.66
	HT	22.69 (5.58)	27.08 (4.13)	36.07 (5.54)	< 0.001	η^2^ = 0.52
	EF	15.19 (9.20)	17.12 (7.15)	27.60 (7.94)	0.0001	η^2^ = 0.29
Victoria Stroop Test	VST I	14.93 (3.03)	14.65 (2.83)	14.80 (3.14)	0.0286	ϵ^2^ = 0.13
	VST II	6.28 (2.42)	8.08 (2.17)	9.00 (2.73)		
	II	2.09 (0.71)	1.65 (0.36)	1.79 (0.39)		

SDMT, Symbol Digit Modalities Test; CTT, Color Trails Test; CTT1, first part of CTT; CTT2, second part of CTT; CTT.II - CTT Interference Index; CVLT, California Verbal Learning Test; CVLT.TRT, Total Recall Trials; CVLT.SDFR, Short-Delay Free Recall; CVLT.LDFR, Long-Delay Free Recall; CVLT.IE, Intrusions Errors; CVLT.TE, Total Errors; CVLT.DI, Discrimination Index; BVRT, Benton Visual Retention Test; NCR, Number of Correct Reproductions; NE, Number of Errors; VFT, Verbal Fluency Test; VFT.PF, Phonemic Fluency; VFT.SF, Semantic Fluency; VFT.VF, Verb Fluency; VFT.LT, Low-difficulty Tasks; VFT.HT, High-difficulty Tasks; VFT.EF, Emotional Fluency; VFT.TS, Total Score; VST, Victoria Stroop Test; VST.I, Dot condition; VST.II, Word condition; VST.III, Interference Index.

In the test assessing the ability to learn and remember verbal material (CVLT), in particular in terms of the number of free recalls after a short delay (CVLT.SDFR), the lowest score was obtained by group 1, significantly different from the highest score for group 2 (*p* < 0.001) and group 3 (*p* = 0.008). In the case of the number of free recalls after a long delay (CVLT.LDFR), the lowest average values were obtained in group 1, which was significantly different from group 2 (*p* = 0.000) and group 3 (*p* = 0.001). The highest average number of intrusions in the CVLT.IE test was significantly higher in group 1 compared to group 2 (*p* < 0.001) and group 3 (*p* = 0.031). In contrast, group 2 had a significantly lower average number of intrusions than group 3 (*p* = 0.009). The lowest discrimination index (CVLT.DI) was observed in group 1, which was significantly lower than in group 2 (*p* < 0.001), and in group 3 (*p* = 0.001), which obtained the highest score. Group 1 also made the most errors (CVLT.TE), significantly more than group 2 (*p* = 0.001) and group 3 (*p* = 0.051).

In the BVRT test, group 1 obtained the lowest average number of correct reproductions (BVRT.NCR), significantly lower than group 2, which obtained the highest score (*p* = 0.002), and group 3 (*p* = 0.008). Group 1 made the most errors (BVRT.NE), significantly more than group 3 (*p* = 0.012) and group 2 (*p* < 0.001), again achieving the lowest score.

In the Verbal Fluency Test, taking into account phonetic fluency, the highest average number of words spoken (VFT.PF) was achieved by group 3, significantly higher than group 1 (*p* < 0.001) and group 2 (*p* < 0.001). Group 3 also had a significantly higher number of verbs spoken (VFT.V) compared to group 1 (*p* < 0.001) and group 2 (*p* < 0.001), which performed the worst. Similar differences were obtained when analyzing the average value of the sum of words from low-difficulty tasks (VFT.LT) and high-difficult tasks (VFT.HT), where again the third group received a significantly higher result than the weakest first group (VFT.LT: *p* < 0.001, VFT.HT: *p* < 0.001) and the second group (VFT.LT: *p* < 0.001, VFT.HT: *p* < 0.001). The results of group 1 also differed significantly from those of group 2 for difficult tasks (*p* = 0.021). In the context of analyzing the number of emotion-related words (VFT.EF), group 3 again generated significantly more words than patients in group 1 (*p* = 0.001) and group 2 (*p* = 0.001).

Significantly different (highest) VST II test completion times in the Word condition approach were achieved by group 1 compared to group 2 (*p* = 0.048) and group 3 (*p* = 0.050).

### 3.4 MRI-Based brain atrophy measures in the identified groups

Analyzing *post-hoc* comparisons of MRI-derived brain atrophy parameters in terms of ventricular enlargement, the most frequent statistically significant differences were also obtained between groups 1 and 2 (Table 6). Group 1 had a significantly higher volume of the third ventricle compared to group 3 (*p* = 0.012). The volume of the left choroid plexus was significantly higher for group 1 compared to group 2 (*p* = 0.003). There were no significant differences on the right side. Statistically significant differences in cerebrospinal fluid volume were demonstrated for groups 1 and 2 (*p* = 0.027). The highest value of white matter hypointensities was characterized by group 1 and was significantly higher than in group 2 (*p* = 0.008).

**Table 6:** Mean brain atrophy measures of individual structures normalized to eTIV, *p*-value for intergroup comparisons (mean ± standard deviation).

Brain structures	Feature	Group 1 (*n* = 16)	Group 2 (*n* = 26)	Group 3 (*n* = 15)	*p*-value	Effect size
Ventricle	3rd	0.00135 (0.00052)	0.00107 (0.0003)	0.00109 (0.00046)	0.0253	η^2^ = 0.13
	4th	0.00169 (0.00063)	0.00131 (0.00032)	0.00133 (0.00035)		
Cerebrospinal fluid		0.00103 (0.00031)	0.00081 (0.00041)	0.00082 (0.00018)	0.0186	ϵ^2^ = 0.14
Choroid plexus	Left	0.00048 (0.00012)	0.00037 (0.00095)	0.00041 (0.00011)	0.0035	η^2^ = 0.19
	Right	0.00052 (0.00011)	0.00045 (0.00012)	0.00044 (0.00013)		
White matter hypointensities		0.00600 (0.00383)	0.00292 (0.00232)	0.00381 (0.00277)	0.0065	ϵ^2^ = 0.19
Thalamus proper	Left	0.00392 (0.00036)	0.00443 (0.00062)	0.00431 (0.00057)	0.0146	ϵ^2^ = 0.14
	Right	0.00386 (0.00036)	0.00429 (0.00049)	0.00421 (0.00060)	0.0218	ϵ^2^ = 0.08
VentralDC	Left	0.00223 (0.00038)	0.00249 (0.00026)	0.00243 (0.00028)	0.0105	η^2^ = 0.17
	Right	0.00224 (0.00033)	0.00249 (0.00025)	0.00242 (0.00026)		
Subcortical gray matter volume		0.03367 (0.00382)	0.03569 (0.00291)	0.03527 (0.00387)		
Cingulate cortex	Anterior	0.00045 (0.00017)	0.00054 (0.00015)	0.00056 (0.00011)		
	Posterior	0.00052 (0.00015)	0.00056 (0.00015)	0.00056 (0.00012)		
Cerebellum white matter	Left	0.00832 (0.00101)	0.00908 (0.00095)	0.00902 (0.00137)	0.0355	ϵ^2^ = 0.12
	Right	0.00813 (0.00135)	0.00868 (0.00088)	0.00876 (0.00113)	0.019	η^2^ = 0.14
Cerebral hemisphere white matter volume	Left	0.13984 (0.02249)	0.15307 (0.01086)	0.15249 (0.01755)		
	Right	0.14008 (0.02048)	0.15194 (0.01088)	0.15133 (0.01771)		
Hemisphere cortex volume	Left	0.14549 (0.01507)	0.15164 (0.00981)	0.14744 (0.01161)		
	Right	0.14672 (0.01493)	0.15107 (0.01007)	0.14768 (0.01160)		
Total gray volume		0.39835 (0.03346)	0.41156 (0.02488)	0.40050 (0.03152)		

In terms of subcortical structure volume, patients classified in group 1 had significantly lower thalamus proper volume on the left side compared to group 2 (*p* = 0.012) and on the right side compared to group 2 (*p* = 0.017). No significant differences were found in the volume of the ventral diencephalon (DC) on the left side. However, on the right side, statistical significance was obtained only for differences between groups 1 and 2 (*p* = 0.008).

In the case of differences between groups in the volume of the anterior and posterior corpus callosum, no statistically significant differences were found between groups (*p* > 0.05).

The cerebellum white matter volume, cerebral hemisphere white matter volume, and hemisphere cortex volume for the left and right sides were also not significantly different between the analyzed groups.

## 4 Discussion

### 4.1 General overview

A clear definition of a patient's cognitive profile, especially in neurodegenerative conditions such as MS, is challenging. Data fusion and mathematical modeling enable the identification of neurocorrelates that, beyond reducing the complexity of the assessment battery, improve our understanding of the features and relationships underlying the patient's status and the trajectory of disease degree. For this purpose, this article presents a novel approach to defining the most distinct neuropsychological features and the profile of patients with MS using machine learning methods.

The selection of characteristics allowed for the identification of the four most differentiating variables. Various methods were used, including analysis of variance, QCD determination, followed by correlation analysis and analysis of the mutual influence of features. This approach enables the definition of four datasets (VarFS_cor, VarFS_MI, QCDFS_cor, and QCDFS_MI), each consisting of various cognitive assessment variables. Each of them was then tested as an input dataset to determine the optimal number of clusters and the stability of the algorithm using Partition Around Medoids, Hierarchical Clustering Methods and k-Means. The best results were achieved for the dataset after feature reduction using variance analysis and mutual information, which was divided into three clusters by PAM (with an average stability of 85%). The determined division allowed for the identification of three new groups of patients, defining their cognitive profiles based on the most differentiating neuropsychological variables—assessments of various cognitive abilities.

The definition of cognitive profiles of patients affected by neurodegenerative diseases is an extremely important and frequently discussed aspect. In work Pourzinal et al. (2020) they used neuropsychological data and methods similar to those described in this article—Within-Sum-of-Squared, Silhouette Score, and later k-Means algorithm—four distinct cognitive phenotypes were defined, determining the degree of impairment in patients with Parkinson's Disease with mild cognitive impairment, depending on the dominance of “frontal,” posterior-cortical, or global impairment. Based on feature reduction using the PCA algorithm, determination of the number of clusters using the elbow method, and unsupervised machine learning (k-means algorithm), in the work of Liang et al. (2024), MS subtypes were determined and provided new information on the links between specific white matter fibers, cognitive abilities and disability levels in MS patients. De Meo et al. (2021) identified five cognitive profiles of MS patients based on neuropsychological and imaging data, taking into account specific domains, which was intended to overcome the limitations of traditional dichotomous classification in multiple sclerosis and has the potential to provide a more meaningful measure of the cognitive status of patients with this disease. In the study by Rimkus et al. (2024) based on neuropsychological data and k-means methods, four clusters of MS patients were identified, differentiated in terms of cognitive disability and the relationship between the severity of cognitive impairment and gray matter atrophy. The analysis by Plow and Gunzler (2022) also provided insight into certain clinical implications for patients with MS. The two latent factors identified allowed for the observation that the measures of severity and impact of mental and physical fatigue, depression, and cognitive impairment reported by the subjects themselves are separate entities, with a comparable model accuracy of 95.8%.

From a clinical perspective, the three patient groups identified in the presented article form a clear continuum–from relatively preserved cognitive functioning (Group 2) through an intermediate profile (Group 3) to a globally impaired phenotype (Group 1). These groups differ in both neuropsychological test performance and the severity of brain atrophy measures, as well as in the level of disability assessed using the EDSS scale. Based on relatively simple, short, and patient-friendly tests, rather than a whole battery of tests, it was possible to identify with a high degree of probability those patients who most likely do not require in-depth neuropsychological and radiological diagnostics, and those whose cognitive functioning is already impaired or is expected to deteriorate in the near future.

Importantly, the groups were defined solely based on a limited set of neuropsychological variables (CVLT Total Recall Trails, VFT Semantic Fluency, CTT1, VST III). Nevertheless, differences between the groups were then consistently observed in the results of the entire test battery and in MRI parameters, which supports their clinical validity.

### 4.2 Group characteristics

The patients in Group 1 presented the most unfavorable clinical and cognitive profile, characterized by the highest mean EDSS score (3.0), the highest number of impaired cognitive domains, and the lowest mean score on the MoCA screening test (23.69 points). Compared to the other two groups, they more frequently showed deficits in visuospatial memory, information processing speed, attentional processes, working memory, access to semantic information, and executive functions, particularly in the use of strategies, utilization of cues, selection of relevant information, and cognitive flexibility. In addition, these patients demonstrated the most significant difficulties in verbal learning (the smallest gain in the number of correctly recalled items in the learning Trails), which translated into poorer performance in both short- and long-term verbal memory, as well as reduced accuracy in the recognition of previously learned material. This profile corresponds to the global impairment phenotype in MS, as described in the literature, which is a generalized, multidomain impairment of cognitive functions (Podda et al., 2024). This pattern is reflected in neuroimaging parameters: in Group 1, the most pronounced features of brain atrophy measures were observed, including greater third ventricle volume, increased cerebrospinal fluid volume, and higher white matter hypointensity values compared with Group 2, suggesting more severe neurodegenerative processes and a greater burden of demyelinating lesions. Furthermore, patients in this group had significantly lower bilateral thalamic volumes and reduced volume of the right ventral diencephalon, which is consistent with previous reports on the key role of subcortical structures, particularly the thalamus, in the pathogenesis of slowed information processing and multidomain cognitive impairment in MS (Houtchens et al., 2007; Mirmosayyeb et al., 2024).

Group 2 represents the opposite pole of this continuum, corresponding to a cognitively preserved phenotype. These patients achieved the lowest mean EDSS score (1.75), indicating a mild degree of motor disability, and demonstrated the best performance on tests of verbal and visual memory, as well as very good results on measures of rapid attention control and processing speed. The number of impaired cognitive domains was the smallest in this group, and mean MoCA scores fell within the normal range. On MRI, patients in Group 2 showed less ventricular enlargement, lower CSF volume, and a lower volume of T1-hypointense lesions compared with Group 1, as well as greater thalamic volume. This pattern is consistent with a clinically relatively stable phenotype, characterized by minimal expression of neurodegenerative changes and preservation of cognitive functions (De Meo et al., 2021).

Patients classified in Group 3 presented an intermediate profile, but with clearly distinct qualitative features. Their level of motor disability (mean EDSS 2.57) was higher than in Group 2 but lower than in Group 1, while simultaneously achieving the best performance on verbal fluency tests. Although verbal fluency tasks may appear relatively simple, they require the simultaneous engagement of multiple cognitive functions and are a good indicator of general cognitive resources (Bąk-Sosnowska and Wyszomirska, 2026). However, the results achieved by individuals in Group 3 do not seem to reflect either faster information processing or above-average verbal memory, but rather high efficiency in semantic knowledge retrieval, lexical access, and executive functions such as cognitive flexibility and strategic search of memory stores. Based on a detailed analysis of the demographic and socio-economic variables, we propose the hypothesis that these competencies may be viewed as an expression of cognitive resources accumulated across the lifespan (Sumowski et al., 2013; Sumowski and Leavitt, 2013). Notably, Group 3 did not include the youngest patients, those with the shortest disease duration, nor those with the least pronounced neurodegenerative changes. For this reason, this group appears particularly interesting in the context of further research on behavioral and psychosocial factors that may buffer against cognitive impairment occurring despite the presence of neuropathological changes. This pattern of cognitive functioning is mirrored in the MRI findings, where indices of brain atrophy and T1-hypointense lesion burden assumed intermediate values in this group, less severe than in Group 1 but more pronounced than in Group 2.

### 4.3 Clinical implication

From a clinical perspective, the identified groups may have direct implications for care planning and management. Patients with a profile corresponding to group 1 should be monitored particularly closely for further deterioration in cognitive function, difficulties with daily functioning, and compliance with therapeutic recommendations and safety concerns (e.g., driving, medication management). In this group, it seems reasonable to refer patients early on to targeted cognitive rehabilitation, family education, and possible modification of care arrangements (e.g., support with instrumental activities of daily living).

In groups 2 and 3, despite the absence of significant cognitive impairment, it is worth implementing preventive measures or measures that strengthen protective factors for cognitive functioning, including: supporting adherence, systematic self-observation of mental and physical condition, physical, social and cognitive activity, taking care of sleep and other activities promoting brain health, and education on risk factors for cognitive decline. Regular screening using simple tools is recommended for this patient group.

Additionally, in group 3, we would suggest neuroimaging monitoring. People in this group may achieve high scores in cognitive function tests and even function well in everyday life, despite the degree of neurodegenerative and demyelinating changes. Therefore, there is a risk of overlooking the progression of brain volume loss, which could potentially be slowed down with treatment. This risk can be minimized by ensuring regular radiological diagnostics, including brain atrophy measures assessments, without the need for frequent neuropsychological evaluations.

An important direction for future research is to integrate the identified cognitive phenotypes with more detailed MRI biomarkers. While the present study focused on global brain volumetry and white matter lesion burden, future studies could investigate whether specific lesion locations, regional patterns of brain atrophy measures, or longitudinal changes in MRI better explain the differences observed between cognitive phenotypes. Such multimodal approaches may improve our understanding of the neuroanatomical substrates underlying cognitive impairment in multiple sclerosis and help identify imaging markers associated with an increased risk of cognitive decline and MS progression. Furthermore, combining neuropsychological profiling with MRI measures can support a more personalized patient stratification and facilitate the earlier identification of individuals who would benefit from closer clinical monitoring or targeted therapeutic interventions. Longitudinal validation of these findings in larger cohorts will be essential to determine the predictive value of the proposed cognitive phenotypes.

### 4.4 Study limitations

It should be emphasized that the presented results come from a single-center study involving a relatively small group of patients, and the analysis used is cross-sectional. This limits the possibility of drawing direct conclusions about the dynamics of transition between phenotypes and their prognostic value for the further course of the disease. Furthermore, although information on education, diagnosis of depression, fatigue syndrome, comorbidities, and treatment used was included, the impact of these moderators on the clinical picture of cognitive functioning in MS was not analyzed in detail. In our opinion, these are important issues for further exploration. In addition, no additional statistical standardization of neuropsychological variables was performed prior to feature selection and clustering. Therefore, differences in the score ranges of individual cognitive measures may have influenced their relative contributions to the clustering procedure. Lastly, it is necessary to validate the proposed model using larger, more homogeneous cohorts and in prospective studies that cover multiple time points. Such a study is planned for the next phase of the project. Although validated tools with proven performance were used for brain structure and MS lesion segmentation, the absence of manual refinement represents a limitation of this study, as residual segmentation errors could have influenced individual measurements. However, because the analyses were performed at the group level in a cross-sectional cohort, the impact on the overall conclusions is expected to be limited.

## 5 Conclusion

The use of feature reduction methods has enabled the identification of a concise and straightforward set of cognitive tests suitable for routine clinical practice, thereby facilitating the detection of patients at increased risk of cognitive impairment without the need for an extensive neuropsychological battery. Unsupervised machine learning algorithms identified several distinct cognitive functioning profiles, ranging from clear deficits to relatively well-preserved functions, which correlated with the severity of neurodegenerative processes. A specific group of patients was identified in whom the relationship between MRI-derived structural changes and cognitive impairment appeared less straightforward. This observation may reflect the influence of cognitive reserve and other factors that modify the clinical manifestations of multiple sclerosis and warrants further investigation. Given the cross-sectional and exploratory nature of the study, the identified profiles should be considered hypothesis-generating rather than definitive clinical phenotypes. Nevertheless, they provide a potentially useful framework for integrating cognitive assessment with structural MRI findings in multiple sclerosis. Future multicenter studies with larger cohorts, external validation, and longitudinal follow-up are needed to determine the stability, generalizability, and prognostic significance of these profiles. Routine cognitive assessment remains an important component of comprehensive patient evaluation and may help identify individuals requiring closer monitoring and further neuropsychological assessment.

## Data Availability

The raw data supporting the conclusions of this article will be made available by the authors, without undue reservation.

## References

[B1] Alirezaei M. Forouzannia S. M. Yarahmadi P. Sahraian M. A. Owji M. Bidadian M. . (2021). Demographic features, behavioral measures, and clinical factors as predictors of cognitive function in patients with multiple sclerosis. Mult. Scler. Relat. Disord. 49:102758. doi: 10.1016/j.msard.2021.102758 33567391

[B2] Angelini L. Buckley E. Bonci T. Radford A. Sharrack B. Paling D. . (2021). A multifactorial model of multiple sclerosis gait and its changes across different disability levels. IEEE Trans. Biomed. Eng. 68, 3196–3204. doi: 10.1109/TBME.2021.3061998 33625975

[B3] Bąk-Sosnowska M. Wyszomirska J. (2026). Cognitive functioning of older women with diverse physical fitness and independence: limitations of dementia screening in primary healthcare. Med. Stud. 41, 123–130. doi: 10.5114/ms.2025.156374

[B4] Benesty J. Chen J. Huang Y. Cohen I. (2009). “Pearson correlation coefficient,” in Noise reduction in speech processing (Berlin, Heidelberg: Springer), 1–4. doi: 10.1007/978-3-642-00296-0_5

[B5] Bonett D. G. (2006). Confidence interval for a coefficient of quartile variation. Comput. Stat. Data Anal. 50, 2953–2957. doi: 10.1016/j.csda.2005.05.007

[B6] Cagol A. Schaedelin S. Barakovic M. Benkert P. Todea R.-A. Rahmanzadeh R. . (2022). Association of brain atrophy with disease progression independent of relapse activity in patients with relapsing multiple sclerosis. JAMA Neurol. 79, 682–692. doi: 10.1001/jamaneurol.2022.1025 35575778 PMC9112138

[B7] De Meo E. Portaccio E. Giorgio A. Ruano L. Goretti B. Niccolai C. . (2021). Identifying the distinct cognitive phenotypes in multiple sclerosis. JAMA Neurol. 78, 414–425. doi: 10.1001/jamaneurol.2020.4920 33393981 PMC7783596

[B8] Filippi M. Bar-Or A. Piehl F. Preziosa P. Solari A. Vukusic S. . (2018). Multiple sclerosis. Nat. Rev. Dis. Primers 4:43. doi: 10.1038/s41572-018-0041-4 30410033

[B9] Fischl B. (2012). Freesurfer. NeuroImage 62, 774–781. doi: 10.1016/j.neuroimage.2012.01.021 22248573 PMC3685476

[B10] Gawda B. Szepietowska E. M. (2021). Test fluencji słownej tfs: podręcznik-wersja dla dorosłych.

[B11] Gierus J. Mosiołek A. Koweszko T. Kozyra O. (2015). The Montreal cognitive assessment 7.2-Polish adaptation and research on equivalency. Psychiatr. Pol. 49, 171–179. doi: 10.12740/PP/24748 25844419

[B12] Han Y. Yu L. (2012). A variance reduction framework for stable feature selection. Stat. Anal. Data Min. 5, 428–445. doi: 10.1002/sam.11152

[B13] Houtchens M. Benedict R. Killiany R. Sharma J. Jaisani Z. Singh B. . (2007). Thalamic atrophy and cognition in multiple sclerosis. Neurology 69, 1213–1223. doi: 10.1212/01.wnl.0000276992.17011.b5 17875909

[B14] Huang L. Zhou X. Shi L. Gong L. (2024). Time series feature selection method based on mutual information. Appl. Sci. 14:1960. doi: 10.3390/app14051960

[B15] Iverson G. L. Ivins B. J. Karr J. E. Crane P. K. Lange R. T. Cole W. R. . (2020). Comparing composite scores for the ANAM4 TBI-MIL for research in mild traumatic brain injury. Arch. Clin. Neuropsychol. 35, 56–69. doi: 10.1093/arclin/acz021 31063188

[B16] Jaworowska A. Stańczak J. Bac I. (2019). Test pamięci wzrokowej bentona BVRT: polskie normalizacje dla dzieci i młodzieży oraz osób dorosłych. Warszawa: Pracownia Testów Psychologicznych Polskiego Towarzystwa Psychologicznego

[B17] Kuraria A. Jharbade N. Soni M. (2018). Centroid selection process using wcss and elbow method for K-mean clustering algorithm in data mining. Int. J. Sci. Res. Sci. Eng. Technol. 4, 190–195. doi: 10.32628/IJSRSET21841122

[B18] Kurtzke J. F. (1983). Rating neurologic impairment in multiple sclerosis: an expanded disability status scale (EDSS). Neurology 33, 1444–1452. doi: 10.1212/WNL.33.11.1444 6685237

[B19] Liang X. Yan Z. Li Y. (2024). Exploring subtypes of multiple sclerosis through unsupervised machine learning of automated fiber quantification. Jpn. J. Radiol. 42, 581–589. doi: 10.1007/s11604-024-01535-1 38409299

[B20] Łojek E. Stańczak J. (2010). Kalifornijski Test Uczenia się Językowego (CVLT): Warsaw: Psychological Test Laboratory of the Polish Psychological Association (Polish).

[B21] Łojek E. Stańczak J. (2012). Kolorowy test połączeń wersja dla dorosłych CTT: polska normalizacja: podręcznik. Warszawa: Pracownia Testów Psychologicznych Polskiego Towarzystwa Psychologicznego

[B22] Mancini M. Giulietti G. Spanò B. Bozzali M. Cercignani M. Conforto S. (2016). “Estimating multimodal brain connectivity in multiple sclerosis: an exploratory factor analysis,” in 2016 38th annual international conference of the IEEE engineering in medicine and biology society (EMBC) (Orlando, FL: IEEE), 1131–1134. doi: 10.1109/EMBC.2016.7590903 28268525

[B23] Mirmosayyeb O. Nabizadeh F. Ghaffary E. M. Panah M. Y. Zivadinov R. Weinstock-Guttman B. . (2024). Cognitive performance and magnetic resonance imaging in people with multiple sclerosis: a systematic review and meta-analysis. Mult. Scler. Relat. Disord. 88:105705. doi: 10.1016/j.msard.2024.105705 38885600

[B24] Montalban X. Lebrun-Frénay, C. Oh J. Arrambide G. Moccia M. Amato M. P. Amezcua L. . (2025). Diagnosis of multiple sclerosis: 2024 revisions of the McDonald criteria. Lancet Neurol. 24, 850–865. doi: 10.1016/S1474-4422(25)00270-4 40975101

[B25] Murtagh F. Contreras P. (2012). Algorithms for hierarchical clustering: an overview. WIREs Data Min. Knowl. Discov. 2, 86–97. doi: 10.1002/widm.53

[B26] Park H.-S. Jun C.-H. (2009). A simple and fast algorithm for K-medoids clustering. Expert Syst. Appl., 36, 3336–3341. doi: 10.1016/j.eswa.2008.01.039

[B27] Plow M. Gunzler D. D. (2022). Disentangling self-reported fatigue, depression, and cognitive impairment in people with multiple sclerosis. Mult. Scler. Relat. Disord. 61:103736. doi: 10.1016/j.msard.2022.103736 35405560

[B28] Podda J. Di Antonio F. Tacchino A. Pedullà L. Grange E. Battaglia M. A. . (2024). A taxonomy of cognitive phenotypes in multiple sclerosis: a 1-year longitudinal study. Sci. Rep. 14:20362. doi: 10.1038/s41598-024-71374-7 39223279 PMC11368960

[B29] Pourzinal D. Yang J. H. J. Byrne G. J. O'Sullivan J. D. Mitchell L. McMahon K. L. . (2020). Identifying subtypes of mild cognitive impairment in Parkinson's disease using cluster analysis. J. Neurol. 267, 3213–3222. doi: 10.1007/s00415-020-09977-z 32535681

[B30] Rimkus C. M. Nucci M. P. Avolio I. B. Apóstolos-Pereira S. L. Callegaro D. Wagner M. B. . (2024). Atrophy patterns in patients with multiple sclerosis with cognitive impairment, fatigue, and mood disorders. Neurology 103:e210080. doi: 10.1212/WNL.0000000000210080 39571119

[B31] Sąsiadek M. Hartel M. Siger M. Katulska K. Majos A. Kluczewska E. . (2020). Recommendations of the Polish medical society of radiology and the Polish society of neurology for a protocol concerning routinely used magnetic resonance imaging in patients with multiple sclerosis. Neurol. Neurochir. Pol. 54, 410–415. doi: 10.5603/PJNNS.a2020.0084 33085075

[B32] Shahapure K. R. Nicholas C. (2020). “Cluster quality analysis using silhouette score,” in 2020 IEEE 7th international conference on data science and advanced analytics (DSAA) (Sydney, NSW: IEEE), 747–748. doi: 10.1109/DSAA49011.2020.00096

[B33] Sinaga K. P. Yang M.-S. (2020). Unsupervised K-means clustering algorithm. IEEE Access 8, 80716–80727. doi: 10.1109/ACCESS.2020.2988796

[B34] Sumowski J. F. Leavitt V. M. (2013). Cognitive reserve in multiple sclerosis. Mult. Scler. J. 19, 1122–1127. doi: 10.1177/1352458513498834 23897894

[B35] Sumowski J. F. Rocca M. A. Leavitt V. M. Riccitelli G. Comi G. DeLuca J. . (2013). Brain reserve and cognitive reserve in multiple sclerosis: what you've got and how you use it. Neurology 80, 2186–2193. doi: 10.1212/WNL.0b013e318296e98b 23667062 PMC3721094

[B36] Thompson A. J. Banwell B. L. Barkhof F. Carroll W. M. Coetzee T. Comi G. . (2018). Diagnosis of multiple sclerosis: 2017 revisions of the Mcdonald criteria. Lancet Neurol. 17, 162–173. doi: 10.1016/S1474-4422(17)30470-2 29275977

[B37] Troyer A. K. Leach L. Strauss E. (2006). Aging and response inhibition: normative data for the Victoria Stroop Test. Aging Neuropsychol. Cogn. 13, 20–35. doi: 10.1080/138255890968187 16766341

[B38] Tsamardinos I. Greasidou E. Borboudakis G. (2018). Bootstrapping the out-of-sample predictions for efficient and accurate cross-validation. Mach. Learn. 107, 1895–1922. doi: 10.1007/s10994-018-5714-4 30393425 PMC6191021

[B39] Wiltgen T. McGinnis J. Schlaeger S. Kofler F. Voon C. Berthele A. . (2024). LST-AI: a deep learning ensemble for accurate MS lesion segmentation. NeuroImage 42:103611. doi: 10.1016/j.nicl.2024.103611 38703470 PMC11088188

[B40] Yousef H. Malagurski Tortei B. Castiglione F. (2024). Predicting multiple sclerosis disease progression and outcomes with machine learning and mri-based biomarkers: a review. J. Neurol. 271, 6543–6572. doi: 10.1007/s00415-024-12651-3 39266777 PMC11447111

[B41] Zawiślak-Fornagiel K. Galus W. Ledwoń D. Wyszomirska J. Romaniszyn-Kania P. Bożek O. . (2026). Cognitive and clinical associations with lesion load and EEG microstate alterations in multiple sclerosis. Comput. Med. Imaging Graph. 131:102766. doi: 10.1016/j.compmedimag.2026.102766 41985397

